# The Bankart repair versus the Putti-Platt procedure: A randomized study with WOSI score at 10-year follow-up in 62 patients

**DOI:** 10.3109/17453670902988345

**Published:** 2009-06-01

**Authors:** Björn Salomonsson, Hassan Abbaszadegan, Suzanne Revay, Ulf Lillkrona

**Affiliations:** Division of Orthopedics, Karolinska Institutet, Danderyd HospitalStockholmSweden

## Abstract

**Background and purpose** This randomized study compared clinical results after surgery for posttraumatic shoulder instability with either an anatomical repair or an older, less anatomical but commonly used method. The less anatomical procedure has been considered quicker and less demanding, but it has been questioned regarding the clinical result. We therefore wanted to compare the clinical outcome of the two different procedures. Our hypothesis was that the anatomical repair would give less residual impairment postoperatively.

**Methods** Patients with anterior posttraumatic shoulder instability were consecutively randomized on the day before surgery to either a Bankart repair using Mitek GI/GII anchors combined with capsular imbrication (B) (n = 33) or a Putti-Platt procedure (P) (n = 33). Follow-up was performed by examination at 2 years and using a self-evaluation score at 10 years.

**Results** At the 2-year follow-up, we found no difference in muscle strength between patients treated with the two surgical methods and there were no statistically significant differences in the Rowe scores (mean 90 units for both groups). Compared to preoperatively, the decrease in external rotation 2 years after surgery was 10 degrees in the P group and 3 degrees in the B group (p = 0.03). 10 years after surgery, 62 of 66 patients replied to a questionnaire sent by mail. It included a self-evaluating quality of life score for shoulder instability (WOSI) for evaluation of their shoulder function. In the P group 15 patients and in the B group 19 patients reported they had experienced either a redislocation or a subluxation with a new feeling of shoulder instability. Mean WOSI score was similar in the P and B groups: 80% and 83%, respectively. The WOSI score was 87% for patients with stable shoulders (n = 28) and 77% for those with unstable shoulders (n =34) (p = 0.005).

**Interpretation** With assessment of pain and general shoulder function, only a small difference was found between the two methods. The WOSI scores for stable shoulders indicated that some shoulders still had impaired function even though the shoulders had become stable.

## Introduction

In 1923, Bankart described the principal pathology of chronic instability after a posttraumatic shoulder dislocation. Although Bankart at that time had already described a technique for anatomical repair of an unstable shoulder, many surgeons have continued to use less anatomical methods (Hovelius et al. 1983, Weber et al. 1984, Toolanen et al. 1990) with good results. One of the most well-known techniques used for shoulder stabilization has been the Putti-Platt method (Osmond-Clarke 1948, Symenoides 1972). The interest in this method has, however, progressively declined over the last 15 years because of the risk of impaired external rotation and a high rate of late osteoarthritis (Konig et al. 1997, Kiss et al. 1998).

We compared the clinical outcome after surgery involving either an anatomical repair (the Bankart method) or an older commonly used method (the Putti-Platt method). Our hypothesis was that the anatomical repair would give less residual impairment.

## Patients and methods

Between November 1991 and April 1995, 66 patients (mean age 28 (16–63) years, 49 men) who had had a history of recurrent anterior posttraumatic shoulder instability (unidirectional) were selected for surgery and were included in this study (Table 1). During this period, altogether 120 shoulders were treated surgically for shoulder instability at the Orthopedic Department of our hospital. Exclusion criteria for the study were prior surgery of the affected shoulder (8 patients), unwilling-ness to participate in the study (5 patients), or arthroscopic treatment (5 patients). 28 other patients were excluded because of atraumatic instability or general joint laxity. In addition, 2 had a posterior instability, 4 had painful occult instability, and 2 were excluded for other reasons.

**Table 1.** Preoperative data and perioperative findingsfor the 2 groups, median (range)

	Putti-Platt (n = 33)	Bankart (n = 33)	p-value
Median age	29 (17–52)	26 (16–63)	0.8
Female / Male	11 / 22	6 / 27	0.2
Duration of instability, months	32 (4–300)	42 (7–144)	0.03
Dominant arm	14 patients	19 patients	0.2
Number dislocations	10 (1–100)	7 (1–50)	0.08
Number subluxations	8 (0–100)	7 (0–100)	0.2
Mean Rowe score preop.	57 units	58 units	0.5
(95% CI)	53–60	55–62	
Glenoid defect / wear	7 / 15	7 / 14	0.6
Bankart lesion, length in mm	20 (0–35)	15 (0–35)	0.3
External rotation after			
wound closure	–7°	18°	< 0.001
(95% CI)	–1° to –13°	12° to 23°	

All patients gave informed consent to participate in this study, which was approved by the Ethics Committee of Karolinska Hospital, Stockholm (nr 92:210) and was conducted in accordance with the Helsinki Declaration of 1975, as revised in 2000. Randomization was performed by opening the next in a series of consecutively numbered sealed envelopes on admission the day before surgery. Each envelope contained information on which procedure was to be performed. This was either a modified Putti-Platt procedure (P group, n = 33) (Symenoides 1991) or a Bankart repair combined with capsular imbrication (B group, n = 33) (Rockwood Jr 1990, Wirth et al. 1996). For the Bankart repair, Mitek GI/GII anchors were used and the surgical procedure was performed by 1 of 3 shoulder surgeons (UL, HA, or BS).

Preoperatively, joint laxity, direction of instability, and range of motion were assessed and a Rowe score was recorded. At the start of the study, the Rowe score (Rowe 1988) was the only specific shoulder instability score available in Swedish, and the Constant score was not considered sensitive enough to evaluate shoulder instability. The Rowe score was therefore chosen for assessment both before surgery and at the 2-year follow-up.

### The Bankart lesion

The so-called Bankart lesion is a detachment of the inferior glenohumeral ligament from the rim of the glenoid, including the anterior-inferior capsule with the labrum and periosteum of the scapular neck. This type of lesion is normally the result of a traumatic anterior shoulder dislocation (Bankart 1938, Moseley 1961). The capsule has usually been stretched or torn, with permanent deformation (Bigliani et al. 1992, McMahon et al. 1999).

### The Bankart procedure

**Figure 1.**
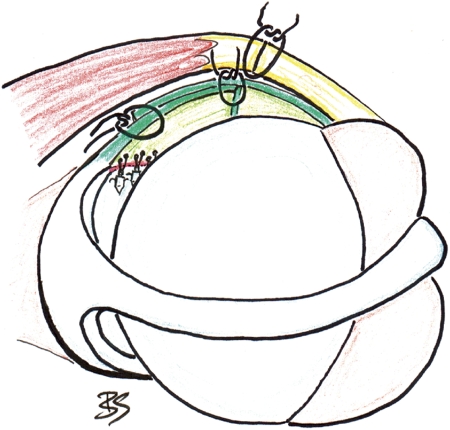
The Bankart repair with Figure 2. The modified Putti-suture anchors and capsular Platt procedure (Symenoides imbrication (Rockwood Jr 1990). 1991). Right shoulder seen from Right shoulder seen from above. above.

The Bankart repair and capsular imbrication (B) was performed as described by Rockwood (Rockwood Jr 1990, Wirth et al. 1996) combined with Mitek suture anchors (Figure 1).

### The Putti-Platt procedure

**Figure 2.**
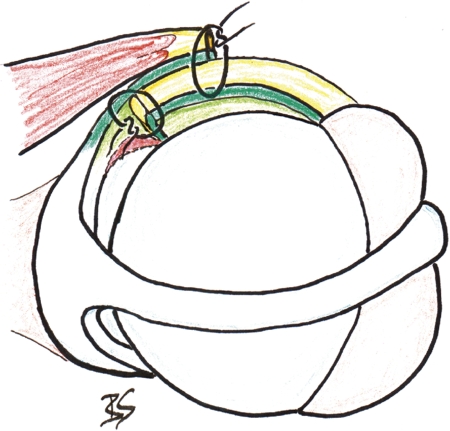
The modified Putti-Platt procedure (Symenoides 1991). Right shoulder seen from above.

Putti and Platt developed this procedure independently of each another in the early 1920s. It was not until 1948 that the procedure was named Putti-Platt when described (Osmond-Clarke 1948). In the present study, the modified technique for Putti-Platt (P) described by Symenoides (1991) was used (Figure 2).

In both groups the postoperative immobilization period was 4 weeks in a shoulder brace, followed by a restricted rehabilitation program for a further 8 weeks, and a return to contact sport 6 months after surgery at the earliest.

More than 2 years after surgery, all 66 patients were re-examined. Muscle strength in abduction and internal rotation was measured with an Isobex. For all patients, the Rowe shoulder score was assessed by the same surgeon (BS). Active and passive range of motion both in sitting and supine position, as well as rotation measured with abducted and adducted arm, were assessed in all patients by an independent physiotherapist (SR). The Rowe score and the measurements were compared to preoperative values, and also against the figures attained for the patient’s unaffected arm. A comparison was then made between the two groups. The definition of recurrent instability was either a complete dislocation or a subluxation described by the patient as a distinct episode of instability, without a dislocation, but with a feeling of unnatural joint movement and fear of an imminent dislocation.

10 years after surgery, the patients were sent a questionnaire by mail asking if they had suffered any subluxation or dislocation of their shoulder. No clinical or radiological examinations were performed at the 10-year follow-up. Patients were also asked if they had experienced any problems with instability in the other shoulder, or if there was any family history of shoulder instability. At the 10-year follow-up we used the WOSI score (Kirkley et al. 1998) to assess patients with shoulder instability. All patients were asked to complete the self-evaluation WOSI questionnaire developed by Kirkley et al. (1998), which gives a quality of life score for patients with shoulder instability. 30 patients in the P group and 32 patients in the B group answered the questionnaire. 1 patient had died and the remaining 3 patients were contacted by telephone, so all the results concerning postoperative shoulder stability were known. The WOSI score was expressed as a percentage of the best possible result, which is defined as the result of normal shoulder function. One of the authors (UL) had translated the questionnaire into Swedish and the Swedish version has been validated and shown to have the same degree of reliability as the original score in English (Salomonsson et.al. 2009).

### Statistics

Student’s t-test was used for testing between groups. In a power analysis, we found that 15 patients in each group would be required in order to give an 80% chance of detecting a difference in mean range of motion of 10 degrees and of 10 points in the Rowe score (SD = 10 degrees/10 points) at a 5% level of significance using the unpaired t-test. The Chi-squared test and the Kruskal-Wallis test were used for non-parametric data. Power analysis for the possibility of detecting a difference between the two surgical methods concerning recurrent instability or for detection of late postoperative osteoarthritis was done before the study began. It was found that a minimum of 80 patients would be required in each group in order to have an 80% chance of detecting a difference of 15% (5% versus 20% for the two groups for both recurrence rate and the incidence of late postoperative osteoarthritis) at the 5% level of significance using the Chi-squared test. We could not hope to recruit that number of patients, and thus it was not a main aim of the study to examine data related to recurrence rate or dislocation arthropathy.

## Results

### At surgery (Table 1)

Of the 66 shoulders, 58 had a Bankart lesion, 14 had a defect of the anterior glenoid (including bony Bankart lesions), 29 had minor glenoid wear, and 33 involved the dominant arm, with no differences between the two groups (Table 1). The median operation time was shorter in the P group, at 74 (45–105) min, than in the B group, at 105 (70–140) min (p < 0.001). External rotation at wound closure was statistically significantly different between the two procedures, with less than neutral (–7°) for the P and 18° for B (p < 0.001).

### 2-year results (Tables 2 and 3)

All 66 patients were examined 2 years after surgery. The post-operative muscle strength in external and internal rotation was not significantly different between the two groups, as measured by an Isobex; nor was there any significant difference in muscle strength between operated and unoperated shoulders.

**Table 2.** Mean differences in outcome at the 2-year follow-up

	Putti-Platt (n = 33)	Bankart (n = 33)	p-value
Decrease in external rotation **^a^**	–10°	–3°	0.03
(95% CI)	–15° to –6°	–7° to 1°	
Active external rotation			
postoperatively	59°	68°	0.03
(95% CI )	54°–65°	62°–73°	
Painful movements	10 patients	1 patient	0.003
Anterior apprehension	15 shoulders	10 shoulders	0.2
Rowe score postoperatively	90 units	90 units	0.9
(95% CI)	86–95	85–94	
Improvement in Rowe score	33 units	31 units	0.8
(95% CI)	27–38	27–38	
Rowe score > 70 points	30 patients	28 patients	0.7
(good and excellent)			
Strength, abduction (kg)	9.6	10.7	0.3
(95% CI)	8.3–11.0	9.3–12.0	
Strength, internal rotation (kg)	11.5	12.1	0.6
(95% CI)	9.8–13.2	10.4–13.8	

**^a^** Mean decrease at 2 years compared to preoperative value.

58 of the 66 shoulders were considered excellent or good (with a Rowe score of > 70 units) at the 2-year follow-up. Subjectively, when questioned 59 patients stated that they had better or much better shoulder function postoperatively, and 2 reported some decrease in function. The mean Rowe score was 90 in both groups. The mean improvement in Rowe score was similar in the two groups; nor was there any difference between the two groups at the 2 years follow-up regarding return to participation in sporting activities at the preoperative level. 6 of the 14 patients with failures at 2-year had only experienced subluxations.

The range of motion differed only in external rotation of the shoulder with the arm in the neutral position, so that compared to preoperatively, the active external rotation at follow-up had decreased by 10 degrees in the P group and by 3 degrees in the B group (p = 0.025). In the P group, the mean external rotation was more restricted compared to preoperatively in the stable group (–14°) than in the unstable group (–1°) (p = 0.03). In the B group there was no significant difference in external rotation between the patients with stable shoulders (–1°) and those with unstable shoulders (–6°). Slight pain during movement was commoner in the P group (p = 0.003), but no patients reported having severe or worse pain after surgery than they had before. At the 2-year follow-up, 2 patients in the P group and 3 patients in the B group had been reoperated—all 5 because of recurrent dislocations.

### 10-year results

Of the 30 patients in the P group who returned the 10-year questionnaire, 15 had either had a redislocation or a subluxation. Of the 32 patients in the B group who returned the questionnaire, 19 had had a redislocation or a subluxation. 1 patient in the P group who had had a reoperation because of recurrent instability had died, but had been assessed during re-operation, and 3 patients were contacted by telephone. There were no statistically significant differences in the outcome regarding sex, glenoid status, or size of the Bankart lesion.

Of the 62 patients who completed the 10-year questionnaire, 19 reported instability problems in the unoperated shoulder. Of the 34 patients who had suffered recurrent instability in the operated shoulder, there were 11 patients with problems in the other shoulder. Of the 28 who had remained stable in the operated shoulder 10 years after surgery, 8 reported problems with instability in the unoperated shoulder.

At the 10-year follow-up there were 8 manifest redislocations in the P group and 11 in the B group. 16 patients in the P group and 19 patients in the B group had experienced subjective instability in the operated shoulder (Table 3). Of these 35 patients, 14 had had stable and well-functioning shoulders for several years after the recurrence. 9 patients in the P group and 12 in the B group had persistent instability problems or had been reoperated because of recurrences. 8 patients underwent reoperations on their shoulders, 4 in each group (1 of these reoperated patients had later died) (Table 4).

**Table 3.** Breakdown of patients in terms of time to failure

Time to failure:	0–2 years (n = 14)	2–5 years (n = 10)	5–10 years (n = 11)
Bankart	8	8	3
Putti-Platt	6	2	8
Redislocations	9	4	6
Subluxations only	5	6	5

**Table 4.** Mean WOSI score (%) and (95% confidence intervals) for the stable shoulders compared with separate groups of unstable shoulders at the 10-year follow-up (n = 62)

	Stable (n = 28)	All unstable (n = 34)	p-value vs. stable	Occasional **^a^** (n = 14)	p-value vs. stable	Recurrent **^b^** (n = 13)	p-value vs. stable	Reoperated **^c^** (n = 7)	p-value vs. stable
Physical	88 (84–92)	81 (78–85)		83 (77–89)		78 (72–85)	0.01	84 (76–93)	
Sport	89 (83–96)	73 (67–79)	< 0.001	80 (70–89)	0.05	72 (62–82)	0.001	62 (49–76)	< 0.001
Lifestyle	88 (81–94)	79 (73–85)		83 (73–93)		74 (63–84)	0.03	81 (67–95)	
Emotion	80 (70–89)	65 (56–74)	0.03	80 (67–92)		46 (33–59)	< 0.001	71 (53–88)	
WOSI Total	87 (82–92)	77 (72–82)	0.005	82 (75–89)		72 (64–79)	0.001	78 (68–88)	

**^a^** Occasional: recurrent on one or more occasions that were associated with subjective instability, but the patients considered their shoulders to be stable and had no further problems for several years.

**^b^** Recurrent on several occasions: the patients considered their shoulder to be unstable.

**^c^** Reoperated: 8 patients, but 1 patient died before the 10-year follow-up.

The mean WOSI score was higher in stable shoulders (87%) than in unstable shoulders (77%) (p = 0.005). There were also differences between the groups in the domains of the WOSI score, especially in Sport and Emotion. The mean WOSI score was 83% in the B group and 80% in the P group (p = 0.4) (Table 5).

**Table 5.** The WOSI score (%) (with individual domains) at the 10- year follow-up. Values are mean (95% confidence intervals). There were no statistically significant differences

	Putti-Platt (n = 30)	Bankart (n = 32)
Physical	83 (78–87)	86 (82–90)
Sport	78 (71–85)	83 (76–90)
Lifestyle	83 (76–89)	83 (77–90)
Emotion	69 (59–78)	74 (65–83)
WOSI Total	80 (75–86)	83 (78–88)

## Discussion

In addition to dislocation or subluxation, there are several other symptoms that may affect the patient’s well-being after shoulder stabilization. At the 2-year follow-up, the P group had more shoulder pain and greater restriction in external rotation than the B group. The decrease in external rotation after Putti-Platt repair was less in our series than has been reported in other studies (Kiss et al. 1998, Pap et al. 1998). This may be a function of the technical modification that we used. This modified procedure has been described by several surgeons (Brav 1955, Symenoides 1972, Leach et al. 1982); the lateral stump of subscapularis tendon is not sutured to the tighter labral structures at the glenoid margin but only overlapped and attached to the capsule, which means that the Bankart lesion is not repaired. The Putti-Platt procedure has been criticized because of a risk of reduced range of motion, and has not been recommended for young patients or for patients with high demands on shoulder function (Regan et al. 1989, Fredriksson and Tegner 1991). Several studies on the Putti-Platt technique have reported recurrence rates of 20% or higher (Hovelius et al. 1979, Varmarken and Jensen 1989, Fredriksson and Tegner 1991, Konig et al. 1997). Pap et al. (1998) presented a 36% recurrence rate at 7-year follow-up. It is interesting to note that in a study by Kiss et al. (1998), the recurrence rate after 9 years was only 9% but there was also a 24-degree reduction in external rotation and as many as one-third of the patients had problems with pain.

The Bankart repair has been considered a demanding procedure (Adams 1948, Norlin 1994, Karlsson et al. 1995), but there have been many reports of good results (Rowe et al. 1978, Hovelius et al. 1979). Suture anchors have been developed and this has made the method technically less demanding (Norlin 1994, Karlsson et al. 1995). Many surgeons combine a Bankart repair with a capsular shift to reduce the amount of capsular tissue and to restore the anatomy (Rowe et al. 1978, Protzman 1980, Rockwood 1990, Norlin 1994, Karlsson et al. 1995).

The recurrence rates reported following a Bankart repair vary. Rowe et al. (1978) reported a recurrence rate of 3% 6 years after surgery. Hovelius et al. (1979) presented a 7-year follow-up with only 2% recurrence. In both of these studies, the capsulolabral complex was fixed to the glenoid with sutures through drill holes. Tamai et al. (1999) performed Bankart repairs using either trans-osseous fixation or with suture anchors, and found a higher risk of redislocation when anchors were used. In a retrospective study comparing the Putti-Platt procedure to a classic Bankart suture, Varmarken and Jensen (1989) found a redislocation rate of 13% in the Bankart group at the 4-year follow-up as compared to 22% in the Putti-Platt group. Many recent reports have shown higher recurrence rates. After having used suture anchors for Bankart repair in open surgery, failure rates of 17% to 30% have been reported (Roberts et al. 1999, Magnusson et al. 2002, Berendes et al. 2007). Other authors have described redislocation rates of between 5% and 10% after open suture anchor repair (Norlin 1994, Karlsson et al. 1995).

The high redislocation rate with both techniques in our study is not easily explained, but it may be related to factors other than the modifications of the procedures we chose. Our patients had a high number of recurrences before surgery, which could influence the outcome (Potzl et al. 2003)—as could the long time from the first dislocation until surgery. Other factors in common with some of the studies with a high recurrence rate are the long follow-up time and an extensive definition of recurrence (Magnusson et al. 2002, Berendes et al. 2007). The degree of shift in the B group and the duplication of the capsule in the P group, measured during surgery as the restriction in external rotation directly after closure of the wound, was judged to be adequate and substantial. The postoperative rehabilitation program was not especially unrestricted, and it seems unlikely that the high rate of recurrence could be due to this.

One possible contribution to the high recurrence rate may be that we found a rather high percentage of bilateral shoulder instability at the 10-year follow-up. A high frequency of bilateral instability has also been reported in other studies (O’Driscoll and Evans 1991, Kiss et al. 1998). However, the high rate in our study is remarkable since we had excluded all patients (23% of all primary stabilizations) with atraumatic instability and excessive joint laxity.

The WOSI quality-of-life score (Kirkley et al. 1998) has a high degree of validity, reliability, and responsiveness to conditions of instability relative to several other scores. The total WOSI score in patients with unstable shoulders was lower than in those with stable shoulders. It is particularly in the domain of emotional well-being that the score is lower in patients with persistent recurrent instability than in those with a stable shoulder (Table 4). The WOSI score was also lower for those with unstable shoulders than for those who were reoperated for recurrent instability. It is not difficult to understand that the WOSI score, which has been designed to address symptoms in instability patients, gives lower ratings for patients with recurrent instability after surgery. It is more interesting that the mean WOSI score in our group with stable shoulders was far from high, and that the patient was to some extent aware of the impairment in quality of life, which is in agreement with other studies (Bottoni et al. 2006, Sachs et al. 2007). One possible explanation may be that the surgical stabilization of an unstable shoulder does not prevent the development of dislocation arthropathy in the long term, as described by Pelet et al. (2006) and by Hovelius et al. (2006).

One weakness of our study was that it did not include radiographs at the 10-year follow-up. The strengths of our study were that it was randomized between two frequently used methods in a selected and well-defined patient group with traumatic anterior recurrent dislocations of the shoulder, that the outcome was measured by a self-evaluation score, that the subluxations were included as recurrences of instability, and that we had a high follow up-rate over 10 years. We believe that evaluation of shoulder stabilization surgery requires long-term follow-up and measurement of outcome regarding quality of life, and not only a description of recurrence rates.

In summary, we found only minor differences between the modified Putti-Platt and the Bankart repair with suture anchors. Some stable shoulders still had impaired function. The range of motion in external rotation was more restricted in the Putti-Platt group.

## References

[CIT0001] Adams JC (1948). Recurrent dislocation of the shoulder.. J Bone Joint Surg (Br).

[CIT0002] Bankart ASB (1923). Recurrent or habitual dislocation of the shoulder joint.. BMJ.

[CIT0003] Bankart ASB (1938). The pathology and treatment of recurrent dislocation of the shoulder joint.. Br J Surg.

[CIT0004] Berendes TD, Wolterbeek R, Pilot P, Verburg H, te Slaa RL (2007). The open modified Bankart procedure: outcome at follow-up of 10 to 15 years.. J Bone Joint Surg (Br).

[CIT0005] Bigliani LU, Pollock RG, Soslowsky LJ, Flatow EL, Pawluk RJ, Mow VC (1992). Tensile properties of the inferior glenohumeral ligament.. J Orthop Res.

[CIT0006] Bottoni CR, Smith EL, Berkowitz MJ, Towle RB, Moore JH (2006). Arthroscopic versus open shoulder stabilization for recurrent anterior instability: a prospective randomized clinical trial.. Am J Sports Med.

[CIT0007] Brav EA (1955). An evaluation of the Putti-Platt reconstruction procedure for recurrent dislocation of the shoulder.. J Bone Joint Surg (Am).

[CIT0008] Constant CR, Murley AHG (1985). A Clinical Method of Functional Assessment of the Shoulder.. Clin Orthop.

[CIT0009] Fredriksson A-S, Tegner Y (1991). Results of the Putti-Platt operation for recurrent anterior dislocation of the shoulder.. Int Orthop.

[CIT0010] Hovelius L, Thorling J, Fredin H (1979). Recurrent anterior dislocation of the shoulder. Results after the Bankart and Putti-Platt operations.. J Bone Joint Surg (Am).

[CIT0011] Hovelius L, Akermark C, Albrektsson B, Berg E, Korner L, Lundberg B, Wredmark T (1983). Bristow-Latarjet procedure for recurrent anterior dislocation of the shoulder. A 2-5 year follow-up study on the results of 112 cases.. Acta Orthop Scand.

[CIT0012] Hovelius L, Sandstrom B, Saebo M (2006). One hundred eighteen Bristow-Latarjet repairs for recurrent anterior dislocation of the shoulder prospectively followed for fifteen years: study II-the evolution of dislocation arthropathy.. J Shoulder Elbow Surg.

[CIT0013] Karlsson J, Järvholm U, Swärd L, Lansinger O (1995). Repair of Bankart lesion with a suture anchor in recurrent dislocation of the shoulder.. Scand J Med Sci Sports.

[CIT0014] Kirkley A, Griffin S, Mclintock H, Ng L (1998). The development and evaluation of a disease-specific quality of life measurement tool for shoulder instability. The Western Ontario Shoulder Instability Index (WOSI).. Am J Sports Med.

[CIT0015] Kiss J, Mersich I, Perlaky GY, Szollas L (1998). The results of the Putti-Platt operation with particular reference to arthritis, pain, and limitation of external rotation.. J Shoulder Elbow Surg.

[CIT0016] Konig DP, Rutt J, Treml O, Hackenbroch MH (1997). Osteoarthritis and recurrences after Putti-Platt and Eden-Hybbinette operations for recurrent dislocation of the shoulder.. Int Orthop.

[CIT0017] Leach RE, Corbett M, Schepsis A, Stockel J (1982). Results of a modified Putti-Platt operation for recurrent shoulder dislocations and subluxations.. Clin Orthop.

[CIT0018] Magnusson L, Kartus J, Ejerhed L, Hultenheim I, Sernert N, Karlsson J (2002). Revisiting the open Bankart experience: a four- to nine-year follow-up.. Am J Sports Med.

[CIT0019] McMahon PJ, Dettling J, Sandusky MD, Tibone JE, Lee TQ (1999). The anterior band of the inferior glenohumeral ligament. Assessment of its permanent deformation and the anatomy of its glenoid attachment.. J Bone Joint Surg (Br).

[CIT0020] Moseley HF (1961). Historical aspects.. Recurrent dislocation of the shoulder. 1 ed..

[CIT0021] Norlin R (1994). Use of Mitek anchoring for Bankart repair: A comparative randomized, prospective study with traditional bone sutures.. J Shoulder Elbow Surg.

[CIT0022] O'Driscoll SW, Evans DC (1991). Contralateral shoulder instability following anterior repair. An epidemiological investigation.. J Bone Joint Surg (Br).

[CIT0023] Osmond-clarke H (1948). Habitual dislocation of the shoulder. The Putti-Platt operation.. J Bone Joint Surg (Br).

[CIT0024] Pap G, Machner A, Nebelung W, Halm JP, Merk H, Grasshoff H (1998). [Long-term outcome of Putti-Platt operation in recurrent traumatic ventral shoulder dislocations].. Zentralbl Chir.

[CIT0025] Pelet S, Jolles BM, Farron A (2006). Bankart repair for recurrent anterior glenohu-meral instability: results at twenty-nine years' follow-up.. J Shoulder Elbow Surg.

[CIT0026] Potzl W, Witt KA, Hackenberg L, Marquardt B, Steinbeck J (2003). Results of suture anchor repair of anteroinferior shoulder instability: a prospective clinical study of 85 shoulders.. J Shoulder Elbow Surg.

[CIT0027] Protzman RR (1980). Anterior instability of the shoulder.. J Bone Joint Surg (Am).

[CIT0028] Regan WD, Webster-bogaert S, Hawkins RJ, Fowler PJ (1989). Comparative functional analysis of the Bristow, Magnuson-Stack, and Putti-Platt procedures for recurrent dislocation of the shoulder.. Am J Sports Med.

[CIT0029] Roberts SN, Taylor DE, Brown JN, Hayes MG, Saies A (1999). Open and arthroscopic techniques for the treatment of traumatic anterior shoulder instability in Australian rules football players.. J Shoulder Elbow Surg.

[CIT0030] Rockwood CA, Rockwood CA, Matsen FA (1990). Anterior glenohumeral instability.. The shoulder.

[CIT0031] Rowe CR, Rowe CR (1988). Evaluation of the shoulder.. The shoulder.

[CIT0032] Rowe CR, Patel D, Southmayd WW (1978). The Bankart procedure: a long-term end-result study.. J Bone Joint Surg (Am).

[CIT0033] Sachs RA, Lin D, Stone ML, Paxton E, Kuney M (2007). Can the need for future surgery for acute traumatic anterior shoulder dislocation be predicted?. J Bone Joint Surg (Am).

[CIT0034] Salomonsson B, Ahlström S, Dalén N, Lillkrona U (2009). The Western Ontario Shoulder Instability Index (WOSI): validity, reliability and responsiveness retested in a Swedish translation.. Acta Orthop.

[CIT0035] Symenoides PP (1972). The significance of the Subscapularis muscle in the pathogenesis of recurrent anterior dislocation of the shoulder.. J Bone Joint Surg (Br).

[CIT0036] Symenoides PP, Watson MS (1991). The Putti-Platt procedure.. Surgical disorders of the shoulder.

[CIT0037] Tamai K, Higashi A, Tanabe T, Hamada J (1999). Recurrences after the open Bankart repair: a potential risk with use of suture anchors.. J Shoulder Elbow Surg.

[CIT0038] Toolanen G, Kjellgren A, Olsson H, Hogstrom B (1990). The Alvik glenoplasty for the unstable shoulder. Modification of the Eden-Hybbinette operation in 66 cases.. Acta Orthop Scand.

[CIT0039] Varmarken JE, Jensen CH (1989). Recurrent anterior dislocation of the shoulder. A comparison of the results after the Bankart and the Putti-Platt procedures.. Orthopedics.

[CIT0040] Weber BG, Simpson LA, Hardegger F (1984). Rotational humeral osteotomy for recurrent anterior dislocation of the shoulder associated with a large Hill-Sachs lesion.. J Bone Joint Surg (Am).

[CIT0041] Wirth MA, Blatter G, Rockwood C (1996). The capsular imbrication procedure for recurrent anterior instability of the shoulder.. J Bone Joint Surg (Am).

